# Effects of High Glucose and Lipotoxicity on Diabetic Podocytes

**DOI:** 10.3390/nu13010241

**Published:** 2021-01-15

**Authors:** Ran Nakamichi, Kaori Hayashi, Hiroshi Itoh

**Affiliations:** Division of Nephrology, Endocrinology and Metabolism, Department of Internal Medicine, School of Medicine, Keio University, 35 Shinanomachi, Shinjuku-ku, Tokyo 160-8582, Japan; ranchino.43@gmail.com (R.N.); hiito@keio.jp (H.I.)

**Keywords:** podocyte, high glucose, lipotoxicity, diabetic nephropathy

## Abstract

Glomerular podocytes are highly differentiated cells that cover glomerular capillaries from the outside and have a characteristic morphology with numerous foot processes. The formation of slit membranes between the foot processes serves as a final filtration barrier for urine filtration from the blood. Podocyte damage causes disruption of the slit membrane, subsequent proteinuria and finally glomerulosclerosis, which is a common pathway in various types of chronic kidney disease (CKD). In recent years, there has been an increase in diabetes, due to rapid lifestyle changes, which is the main cause of CKD. Therefore, understanding the effect of diabetic status on podocytes is of great importance to establish a strategy for preventing CKD progression. In this review, we summarize altered glucose and lipid metabolism in diabetic podocytes and also discuss the reversibility of the changes in podocyte phenotype.

## 1. Introduction

Chronic kidney disease (CKD), defined as evidence of structural or functional renal impairment for 3 or more months, is generally progressive and irreversible. The global prevalence of diabetes mellitus (DM) has increased over the past few decades, mainly driven by an increase in the prevalence of type 2 diabetes mellitus (T2DM) due to obesity and the metabolic syndrome [1,2]. Microvascular changes within the kidney often lead to chronic kidney disease, an entity referred to as diabetic nephropathy (DN) [3]. DN is the most common cause of CKD and end-stage kidney disease worldwide [4].

It has recently become clear that the initial glomerular injury affects the podocytes as important target cells for the progression of CKD and end-stage kidney disease [5]. Podocytes are highly differentiated epithelial cells that cover the outer layer of the glomerular basement membrane. Podocytes serve as the final barrier to urinary protein loss through the special formation of foot processes and an interposed slit diaphragm. As podocytes are terminally differentiated cells without a capacity for proliferation or replenishment, chronic injury causes phenotypical changes, detachment and apoptosis in podocytes, leading to disruption of the slit membrane and finally glomerulosclerosis [6,7].

Therefore, understanding podocyte phenotype and function in diabetes is necessary for CKD management and for considering future therapeutic targets. This review summarizes podocyte injury in diabetes, focusing on not only the effect of high glucose itself, but also lipotoxicity that is frequently associated with diabetes.

## 2. Effects of Hyperglycemia on Podocytes in Diabetic Nephropathy (DN)

Hyperglycemia induces a podocytopathy, characterized by cellular hypertrophy, foot process effacement, and podocyte depletion. It is suggested that podocytes may respond to injurious stimuli in different ways, including hypertrophy, dedifferentiation, detachment and depletion, depending on the severity and duration of the injury [8]. Some of the new mechanisms that drive the effects of hyperglycemia on podocytes and possible therapeutic targets are discussed below, divided into chapters by types of podocyte morphological changes, because the function of podocytes primarily depends on their structure and morphology. The key molecules in the impact of hyperglycemia on podocytes in DN are summarized in Table 1.

Glycemic control is the main determinant to prevent the progression to overt DN. Improvement in HbA1c decelerates glomerular filtration rate (GFR) loss and delays the onset of End stage renal diseases (ESRD) in patients with type 1 diabetes mellitis (T1DM) and proteinuria [9]. Moreover, 10 years after pancreas transplantation in type 2 diabetes mellitus (T2DM) under normoglycemia, glomerular lesions were markedly improved, which suggests that the lesions of diabetic nephropathy are reversible and that the kidney can undergo remodeling upon long-term normoglycemia [10]. On the other hand, large clinical trials of diabetes have suggested that even transient treatments or injuries could have a sustained effect on the onset or progression of complications for longer periods, which is known as ‘metabolic memory’ [11,12,13,14]. One of possible mechanisms of the memory is epigenetic alteration. Recent studies indicate that specific DNA methylation of blood or kidney cells in diabetic patients may be associated with development of the nephropathy [15,16,17]. Therefore, better understanding of epigenetic regulation in podocytes is necessary when considering the reversibility or plasticity of podocyte damage following therapeutic approaches in DN. Epigenetic alterations in diabetic podocytes are also described.

### 2.1. Podocyte Hypertrophy

Previous studies of animal models and humans have established that podocyte hypertrophy is associated with the development of DN [18,19]. Angiotensin Ⅱ has been shown to increase the expression of parathyroid hormone-related protein (PTHrP), Transformin Growth Factor β1 (TGF-β1), and cell cycle regulatory protein-p27Kip, which promotes the aggravation of podocyte hypertrophy in high-glucose conditions [20]. Several studies have suggested that mTORC1 (mechanistic target of rapamycin signaling complex 1), a kinase that senses nutrient availability, was upregulated in podocyte of diabetic mice, and closely associated with the activation of podocyte hypertrophy induced by high glucose [21]. Inoki et al. have reported that abnormal mTORC1 activation caused mislocalization of slit diaphragm proteins and induced endoplasmic reticulum (ER) stress in podocytes, which suggest mTORC1 activation in podocytes is a critical event in inducing DN [22]. Moreover, knockout of Ragulator component p18, recruiting of mTORC1 to lysosomal membranes, attenuated its activation and cell injury under diabetic conditions. This points to mTOR-lysosome recruitment as a potential therapeutic target for the treatment of DN [23].

### 2.2. Foot Process Effacement

Foot process effacement is a cytoskeletal rearrangement of podocytes reflected by flattening, widening, and retraction of foot processes that signifies podocyte injury and weakening of the integrity of the glomerular filter barrier, thereby leading to albuminuria in DN [24,25]. Dysregulation of nephrin, an essential transmembrane protein in the slit diaphragm complex [26,27], is an important mechanism of foot process effacement. The expression of nephrin is decreased in DN, resulting in aberrant rearrangement of actin and breakdown of the slit diaphragm and foot process effacement [28]. Mislocalization of nephrin has been also observed in DN [29]. Several studies have suggested that protein kinase C α-type (PKCα) was upregulated under hyperglycemia and mediates beta-arrestin2-dependent nephrin endocytosis [30,31]. Protein kinase C and casein kinase substrate in neurons protein 2(PACSIN2) has also been reported as a molecule involved in nephrin endocytosis [29]. Endpcytosis of nephrin is a promising target molecule for podocyte protective therapy in DN.

**Table 1:** Key molecules associated with high glucose in podocytes.

Key Molecules	Effect on Podocytes	Mechanism	Experimental Model	Expression	Ref.
**mTORC1**	Hypertyophy Foot process effacement GBM thickening podocyte loss	Mislocalization of nephrin, ER stress	db/db mice	increase	[21,22]
**PKCα**	Foot process effacement	Endocytosis of nephrin	STZ mice DN, human	increase	[30,31]
**Integrinα3β**	Podocyte detachment	connecting podocytes with the ^7^GBM	STZ rats DN, human	decrease	[32,33]
**NOX4**	Foot process effacement	ROS production	ApoE(−/−) mice	increase	[34]
**NOX5**	Foot process effacement GBM thickening	ROS production	DN, human	increase	[35]
**TRPC5**	Foot process effacement Podocyte loss	cytoskeletal rearrangement	Dhal SS rats	increase	[36,37]
**TRPC6**	Podocyte apoptosis	intracellular Ca(2+) overload, NOX4-derived ROS production	Dhal SS rats	increase	[38]
**Dnmt1**	Foot process effacement	DNA methylation in the nephrin promoter region	db/db mice	increase	[39]
**KLF4**	Foot process effacement	DNA methylation in the nephrin promoter region	db/db mice DN, human	decrease	[40,41]
**KAT5**	Foot process effacement	Impaired DNA repair	db/db mice	decrease	[42]

mTORC1: mechanistic target of rapamycin complex1, PKCα: Protein kinase Cα, NOX4: nicotinamide adenine dinucleotide phosphate oxdase4,NOX5: nicotinamide adenine dinucleotide phosphate oxdase5, TRPC5: Transient receptor potential cation channel, subfamily C, member 5, TRPC6: Transient receptor potential cation channel, subfamily C, member 6, Dnmt1: DNA (cytosine-5)-methyltransferase 1, KLF4: Krüppel-like transcription factor 4, GBM: glomerular basement membrane, ER: endoplasmic reticulum, ROS: Reactive Oxygen Species, STZ: Streptozocin, DN: diabetic nephropathy, ApoE(−/−): apo E deficiency, Dhal SS: Dhal salt sensitive.

The reduction of nephrin expression by DN results in not only foot process effacement but also insulin signaling alternation, because the cytoplasmic tail of nephrin is necessary for proper insulin signaling [43]. Mice with podocyte-specific deletion of the insulin receptor develop significant albuminuria together with histological features that recapitulate DN in a normoglycemia, which reveals insulin signaling to the podocyte is critical for kidney function [8].

### 2.3. Podocyte Detachment and Apoptosis

The podocyte and glomerular basement membrane (GBM) are closely connected to prevent proteinuria by sustaining the glomerular filtration barrier. Integrin α3β is an important receptor that can tightly connect podocytes with the GBM. The expression of α3β1 has been shown to be decreased in patients with diabetes and in rats with streptozotocin-induced diabetes, contributing to detachment of podocytes from the GBM [32,33]. Podocyte apoptosis is caused by glomerular hyperfiltration as well as hyperglycemia itself. Several studies have shown that TGF-β induces apoptosis in podocytes by stimulating mitogen-activated protein kinase (MAPK) p38 signaling and the classic effector caspase-3 pathway in DN [44,45]. Podocyte apoptosis under high-glucose conditions is associated with the release of mitochondrial and plasma membrane reactive oxygen species (ROS) that trigger the p38MAPK and nicotinamide adenine dinucleotide phosphate oxidases (NOX) signaling pathways [46,47]. In the rodent kidney, three isoforms of the catalytic subunit of nicotinamide adenine dinucleotide phosphate (NADPH) oxidase are expressed (NOX1, NOX2, and NOX4). Nox4 is the main source of renal ROS in a mouse model of DN [34]. NOX5 has been recently reported to be upregulated in human DN podocytes, and alter filtration barrier function through the production of ROS [35]. ROS has obtained increasing attention in recent years as a therapeutic target for DN, as we know bardoxolone methyl, an oral antioxidant inflammation modulator, improves renal function in patients with advanced CKD and T2DM [48]. Several reports have shown that NOX inhibition in vivo reduces albuminuria and podocytopenia in models of diabetes [49,50]. Furthermore, pharmacological inhibition of NOX1 and -4 reduces albuminuria and slows DN progression in a T2DM model [51]. Transient receptor potential canonical 6 (TRPC6) channel is reported to play a critical role on podocyte ROS. High glucose levels have been shown to induce podocyte apoptosis by stimulating TRPC6 channel-mediated elevation of intracellular calcium in the presence of elevated ROS levels [52]. Recent studies demonstrated that angiotensin II enhances albuminuria by activating TRPC6 channels in podocytes, and this pathway is required the production of ROS [53,54]. Furthermore, it has been reported that NOX4-derived ROS is associated with TRPC5/TRPC6 channels [38]. In regard to TRPC5, TRPC5 is activated by Rac-1, which induces podocyte damage [36]. AC1903, A small-molecule inhibitor of TRPC5, suppressed severe proteinuria and prevented podocyte loss in a rat model of hypertensive proteinuric kidney disease, which indicates TRPC5 inhibitors may be valuable for the treatment of progressive kidney diseases [37].

### 2.4. Epigenetic Regulation in Diabetic Podocytes

Recently, the link between altered gene expression and epigenetic regulation has attracted much attention in CKD, especially that due to DN. Previously, we showed increased DNA methylation at the nephrin promoter region in podocytes of murine models of DN and patients with DN, which is associated with decreased expression of the transcription factor Kruppel-like factor 4 (KLF4) [40,41]. Zhang et al. also reported a role of DNA methylation in the nephrin promoter region by DNA methyltransferase (DNMT) 1, which may be a possible target for the treatment of DN [39]. Regarding histone modifications, it has been reported that hyperglycemia causes direct modification of histone acetylation status [55]. Interestingly, Rizotte et al. demonstrated that altered histone acetylation and methylation in diabetic podocytes were sustained even after correction of glycemic control to the normal range by continuous insulin administration [56]. This result indicates the possibility that persistent epigenetic changes in podocytes may contribute to ‘metabolic memory’, which is the persistent effect of transient treatments or injuries on disease progression. These results indicate the possibility that podocyte phenotype may be recovered if altered epigenetic marks could be reversed. Previously we have reported that podocyte phenotype could be recovered by transient restoration of KLF4 expression in podocytes using the Tet-on system, with reversed DNA methylation status [41]. Up to now, it is still unclear the intensity and duration of treatment which is necessary to cause epigenetic recovery in podocytes. Further study is needed to investigate the reversibility and plasticity of the podocyte epigenome in each stage of DN.

### 2.5. DNA Damage and Hyperglycemia

We have discussed epigenetic modifications and the development of CKD, but the process of formation of such epigenetic changes has remained unclear. We have recently focused on the involvement of DNA damage repair in DNA methylation changes in podocytes, since they are terminally differentiated cells without DNA replication. A DNA repair factor lysine acetyltransferase 5 (KAT5) was found to be essential for the maintenance of the podocyte genomic integrity and hyperglycemia was shown to decrease the expression of KAT5 as well as to increase DNA damage in podocytes. Therefore, both of these induce DNA methylation changes in podocytes, which may be involved in the pathogenesis of diabetic nephropathy. [42]. This provides a novel possibility linking podocyte DNA damage to epigenetic changes in metabolic diseases [57]. In humans, it is feasible to evaluate podocyte DNA damage and the expression of DNA methylation modulators using urine-derived cells in patients with diabetes and/or hypertension [58]. Thus, podocyte DNA damage and DNA methylation may be a hopeful target of a diagnostic marker as well as a novel therapy for conquering against DN. When considering epigenetic regulation as a target for DN treatment, such as DNMT1, the problem of side effects always arises. Because DNA damage repair system is specific to cell types [57], factors associated with DNA damage repair may be a hopeful therapeutic target regulating epigenetic status. Future studies are necessary to investigate how to induce epigenetic changes only in the target cells.

## 3. Effects of Lipotoxicity on Podocytes in DN

Hyperglycemia induced metabolic alterations play critical roles in disease initiation, but a cluster of factors, including dyslipidemia and hypertension, could play a role in inducing onset and progression of DN [59]. Evidence has been accumulated to suggest that dyslipidemia is one of the risk factors for progression and regression of diabetic nephropathy [60,61,62,63]. Reduced plasma triglyceride levels in T2DM patients with treatment of fenofibrate result in reduction of albuminuria [64]. Improvement of elevation of serum low-density lipoprotein (LDL)-cholesterol with treatment was associated with an improvement in annual changes in estimated glomerular filtration rate (eGFR) [65]. These observations support the notion of lipid-lowering therapies could provide beneficial effects on dyslipidemia-mediated kidney injury.

Birnkkoetter et al. reported that in mice, podocytes rely primarily on anaerobic glycolysis to maintain glomerular filtration barrier and are relatively insensitive to defect in mitochondrial biogenesis during ischemia damage [66]. Instead, as lipid accumulation is commonly observed in patients with CKD, podocytes are rather sensitive to cellular lipid-mediated glomerular injury [67]. One evolving area of research has focused on the role of lipotoxicity in podocyte damage in DN. Lipotoxicity, which is a disruptive process caused by lipid accumulation in non-adipose tissue, resulting in cell damage and cell death, is closely associated with the pathogenesis of these diseases. Intracellular lipid accumulation causes insulin resistance, the production of reactive oxygen species (ROS) and endoplasmic reticulum stress, all of which could cause renal damage. Several molecules have been reported to be involved in the effects of lipid accumulation on podocytes as described below Table 2 summarizes the key molecules involved in cholesterol and free fatty acids accumulation separately.

### 3.1. Cholesterol Accumulation and Podocytes

The accumulation of cholesterol in podocytes has been shown in diabetic nephropathy (DN) to be involved in the development of glomerular sclerosis [77,78,79]. In addition to DN, clinical studies have shown a similar accumulation of cholesterol in patients with atherosclerosis and focal segmental glomerulosclerosis (FSGS) [68,80,81]. ATP-binding cassette transporters, such as ATP-binding cassette subfamily A member 1 (ABCA1) and ABC subfamily G member 1 (ABCG1), have important roles in cholesterol efflux to high-density lipoprotein (HDL) acceptors and have recently been noted for their association with CKD. The expression of ABCA1 is reduced in type 1 diabetic mice [69]. Similarly, in type 2 diabetic mice, the downregulation of ABCA1 and ABCG1 has been reported, leading to lipid accumulation [68]. Recently, Ducasa et al. have reported that podocyte-specific deletion of ABCA1 rendered mice susceptible to DKD and the accumulation of mitochondrial cardiolipin, and in mice with DN, an increase in cardiolipin oxidation was observed and a cardiolipin peroxidase inhibitor treatment reversed DKD progression, with improvements in podocyte number [82]. The involvement of the renin-angiotensin system (RAS) in cholesterol metabolism in podocytes has been extensively studied. Angiotensin Ⅱ has been reported to induce the accumulation of cholesterol in mouse podocytes, which was related to the expression of genes including ABCA1 and ABCG1 [83]. In addition, we have also reported that in a mouse model of hyperlipidemia, treatment with high-dose angiotensin receptor blockers (ARBs) reduced cholesterol accumulation in the glomerulus and improved proteinuria. The suggested mechanism might involve reduced expression of biglycan, a lipid-retaining proteoglycan, and ACAT1, which converts cholesterol to ester, resulting in a relative increase in free cholesterol for lipid release [70]. Although an important role of RAS in CKD development and progression is widely recognized, it would be of great interest to understand the effect of activated RAS on CKD, focusing on lipid accumulation.

### 3.2. Free Fatty Acids (FFAs) and Triglycerides

Triglycerides (TGs) are lipids that form lipid droplets, and free fatty acids (FFAs) accumulate intracellularly as TGs. Increased uptake of FFAs in podocytes has been observed in DN [79].

CD36 is a multifunctional transmembrane glycoprotein that acts as a transporter for FFA uptake in the kidney, where it plays a main role in FFA uptake in tubular epithelial cells, podocytes and mesangial cells, and is also important in the development of CKD [84]. CD36 is elevated in both mouse models and in humans in the presence of kidney damage [85]. CD36 has been shown to be associated with lipid accumulation in patients with diabetes [71,72]. In addition to DN and in Alport model mice, lipid accumulation with elevated CD36 has been recognized [73]. In podocytes, CD36 mediated FFA uptake increases ROS, leading to apoptosis [86,87,88]. In addition to CD36, other transporters for FFA uptake include fatty acid transport protein 4 (FATP4) and fatty acid-binding protein (FABP). FATP4 is upregulated in podocytes in a diabetic model and is involved in TG accumulation and cell damage [75]. H-FABP has been reported to be expressed specifically in podocytes and is associated with proteinuria in a mouse model of diabetes and in patients with obesity-related nephropathy [76].

### 3.3. Ketone Bodies and Mechanistic Target of Rapamycin Signaling Complex 1 (mTORC1)

Ketone bodies are produced from fatty acid degradation in the liver in the state of glucose depletion, such as fasting, and are known to be an important alternative energy source that is quickly utilized in place of glucose in other tissues. In recent years, ketone bodies have also attracted attention for their role as mediators of nutrient signals in cells [89]. One of the nutrient-sensing signals is mTORC1, which is upregulated in podocytes and tubular cells in mouse models of diabetes and in patients with DM [22,90]. Administration of ketones to a mouse model of diabetes suppressed mTORC1, decreased proteinuria, and prevented podocyte damage. Sodium glucose cotransporter 2 (SGLT2) is known to increase the blood levels of ketones, and the renoprotective effects of SGLT2 have been reported to be associated with elevated ketones and inhibition of mTORC1 [91]. In addition to DN, ketone administration produces similar changes in 5/6 nephrectomized mice and polycystic kidney disease (PKD) model mice, which indicates that time-restricted feeding and ketogenic diets that promote ketosis attenuate mTORC1 signaling and inhibit PKD progression [92].

## 4. Summary

**Figure 1:**
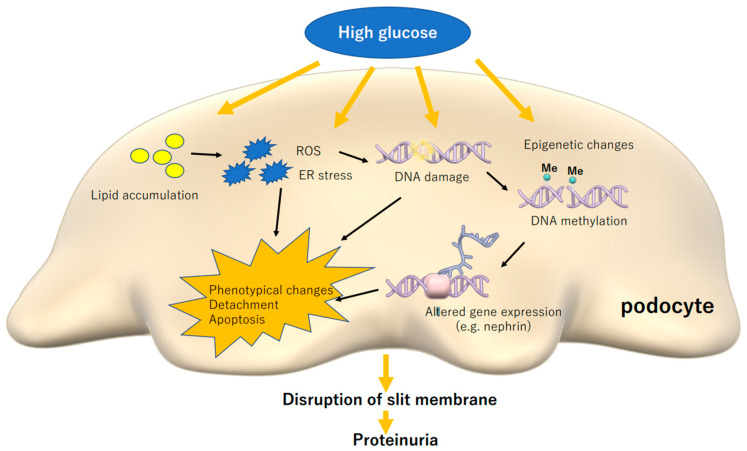
High glucose and induced lipotoxicity causes stress reactions in podocytes, which can cause phenotypical changes, detachment and apoptosis, leading to disruption of slit membrane and proteinuria. ROS: reactive oxygen species, ER: endoplasmic reticulum.

This review summarizes podocyte damage caused by high glucose and lipotoxicity in diabetes as shown in Figure 1. Recent advances in research techniques such as single-cell analysis and epigenetic analysis, are revealing the biology of podocytes in detail. As a characteristic feature of glomerular podocytes, their damage is directly linked to kidney dysfunction and renal prognosis. Epigenetic alteration in podocytes may be important to discuss the reversibility of podocyte phenotype following therapeutic intervention. Understanding metabolic alterations in diabetic podocytes is important to investigate novel strategies for the treatment of ever-increasing DN.

## Figures and Tables

**Table 2:** Key molecules associated with lipotoxicity in podocytes.

Key Molecules	Effect on Podocytes	Mechanism	Experimental Model	Expression	Ref
**ABCA1**	Cholesterol accumulation	efflux of cholesterol	NOD mice T2DM human	decrease	[68,69]
**ABCG1**		efflux of cholesterol	T2DM human	decrease	[68]
**ACAT1**		convert cholesterol to ester	SHL mice	decrease	[70]
**CD36**	FFA accumulation	Transporter of FFA	HFD mice SHL mice AS mice T2DM human	increase	[71,72,73,74]
**FATP4**		Transporter of FFA	ORG human	increase	[75]
**FABP**		Transporter of FFA	db/db mice	increase	[76]

ABCA1: ATP-binding cassette subfamily A member 1, ABCG1: ATP-binding cassette subfamily G member 1, ACAT1: Acetyl-Coenzyme A acetyltransferase 1, FATP4: fatty acid transport protein 4, FABP: Fatty Acid-Binding Protein, FFA: free fatty acids, NOD: Non-obese diabetic, SHL: apo E deficiency spontaneously hyperlipidemic, HFD: high-fat diet feeding, AS: Alport syndrome, ORG: obesity-related-glomerulopathy.

## Data Availability

All data generated during this study are included in this published article.
